# Making Radiomics More Reproducible across Scanner and Imaging Protocol Variations: A Review of Harmonization Methods

**DOI:** 10.3390/jpm11090842

**Published:** 2021-08-27

**Authors:** Shruti Atul Mali, Abdalla Ibrahim, Henry C. Woodruff, Vincent Andrearczyk, Henning Müller, Sergey Primakov, Zohaib Salahuddin, Avishek Chatterjee, Philippe Lambin

**Affiliations:** 1 The D-Lab, Department of Precision Medicine, GROW—School for Oncology, Maastricht University, Maastricht, Universiteitssingel 40, 6229 ER Maastricht, The Netherlands; a.ibrahim@maastrichtuniversity.nl (A.I.); h.woodruff@maastrichtuniversity.nl (H.C.W.); s.primakov@maastrichtuniversity.nl (S.P.); z.salahuddin@maastrichtuniversity.nl (Z.S.); a.chatterjee@maastrichtuniversity.nl (A.C.); philippe.lambin@maastrichtuniversity.nl (P.L.); 2 Department of Radiology and Nuclear Medicine, GROW—School for Oncology, Maastricht University Medical Center+, P.O. Box 5800, 6202 AZ Maastricht, The Netherlands; 3 Department of Medical Physics, Division of Nuclear Medicine and Oncological Imaging, Hospital Center Universitaire de Liege, 4000 Liege, Belgium; 4 Department of Nuclear Medicine and Comprehensive Diagnostic Center Aachen (CDCA), University Hospital RWTH Aachen University, 52074 Aachen, Germany; 5 Institute of Information Systems, University of Applied Sciences and Arts Western Switzerland (HES-SO), rue du Technopole 3, 3960 Sierre, Switzerland; vincent.andrearczyk@hevs.ch (V.A.); henning.mueller@hevs.ch (H.M.)

**Keywords:** radiomics, harmonization, feature reproducibility, deep learning, medical imaging

## Abstract

Radiomics converts medical images into mineable data via a high-throughput extraction of quantitative features used for clinical decision support. However, these radiomic features are susceptible to variation across scanners, acquisition protocols, and reconstruction settings. Various investigations have assessed the reproducibility and validation of radiomic features across these discrepancies. In this narrative review, we combine systematic keyword searches with prior domain knowledge to discuss various harmonization solutions to make the radiomic features more reproducible across various scanners and protocol settings. Different harmonization solutions are discussed and divided into two main categories: image domain and feature domain. The image domain category comprises methods such as the standardization of image acquisition, post-processing of raw sensor-level image data, data augmentation techniques, and style transfer. The feature domain category consists of methods such as the identification of reproducible features and normalization techniques such as statistical normalization, intensity harmonization, ComBat and its derivatives, and normalization using deep learning. We also reflect upon the importance of deep learning solutions for addressing variability across multi-centric radiomic studies especially using generative adversarial networks (GANs), neural style transfer (NST) techniques, or a combination of both. We cover a broader range of methods especially GANs and NST methods in more detail than previous reviews.

## 1. Introduction

Medical imaging is routinely used in clinical practice to assist the decision-making process for diagnostic and treatment purposes [1,2]. Radiomics is an emerging field within medical image analysis that goes beyond qualitative assessment by extracting a large number of quantitative image features [3,4]. The radiomic hypothesis postulates that the quantitative study of medical image data can provide complementary knowledge in a quick and reproducible manner to support clinicians in their decision-making process, assisted by automated or semi-automated software [5,6]. The information acquired can help advance the clinical decision support systems to connect the link between radiomic features and clinical endpoints by building diagnostic, prognostic, and predictive analysis models. Radiomics is the consequence of many decades of computerized diagnosis, prognosis, and treatment research [7,8]. A powerful radiomics approach involves the extraction of various quantitative features from medical images, storing this data in a federated form of a database [9] where several individual databases function as an entity, and the successive mining of data to acquire relevant clinical outcomes [10]. Large quantities of data are required to develop robust predictive models and this amount of data is usually obtained from multiple hospitals and/or institutions. Furthermore, due to the continuous improvement in scanner and protocol settings, this type of data is a moving target. To compensate for the effects scanner/protocol variability might have on the predictive models, large quantities of data are needed to make systems generalize. In these cases, federated (or distributed) learning could be adapted to allow sharing of data between hospitals/institutes to develop robust predictive models [10]. Major management problems still exist even though there are databases that are collecting and cross-referencing massive amounts of radiomics information in addition to other related patient data from millions of case studies [11,12,13,14].

**Figure 1:**
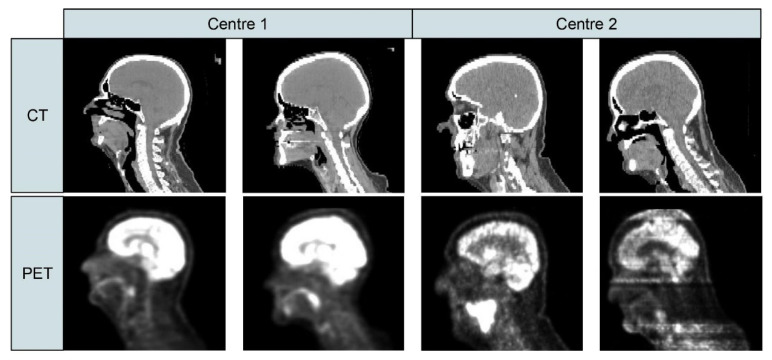
PET and CT slices obtained from two different centers (Center 1 = Centre Hospitalier Universitaire de Sherbrooke, Sherbrooke, Canada and Center 2 = Hôpital Maisonneuve-Rosemont, Montréal, Canada). The top row shows CT images while the bottom row shows PET images. The four columns indicate four different patients. Adapted from [23].

Radiomic feature extraction can be categorized into two main approaches: hand-crafted (derived from traditional statistical and computer vision methods) and deep learning (DL). Hand-crafted radiomics characteristics (such as texture, shape, intensity) provide information on the particular area of the medical imaging scan, often referred to as the region or volume of interest (ROI or VOI), which could be a tumor, a tissue, or an organ as a whole [15]. DL is also a data-driven method that is inspired by the biological neural networks in the human brain. The difference between hand-crafted and DL approaches mostly lies in the way visual representations are learned. For example, some DL algorithms learn complex visual features and perform ROI segmentation using cascading layers with non-linearities by using ‘sliding’ kernels in convolutional neural networks (CNN), while hand-crafted features represent the spatial appearances (texture and shape) by mathematically extracting spatial distribution on inter-pixel relationships, signal intensities, gray-scale patterns, and spectral properties [16]. DL has the benefit of not necessarily requiring prior segmentation masks of the medical imaging scan. However, DL is a ’black box’ approach, i.e., the lack of interpretability of the models and the deep features generated are seen as a key limitation in clinical applications [17]. DL also requires a larger amount of data and/or pre-trained models often trained on diverse domains (e.g., photographic images), in order to perform efficiently and effectively. The vast majority of published radiomic models lack consistent evaluation of performance, sufficient large-scale annotated datasets for radiomic studies, reproducibility, clinical efficacy, and large-scale validation on sufficiently large cohorts, despite these being prerequisites for clinical translation [18,19]. Furthermore, there is a lack of reproducibility of radiomic features while translating results into clinical practice [20]. Ideally, the features extracted using radiomics represent imaging biomarkers and should be independent of image acquisition parameters or protocols [21]. For example, if a patient is scanned in different hospitals, the quantitative features extracted from all these scans should either have similar values or the correct transformation should be known. Scanner protocols and hardware are constantly changing over time and differ across hospitals. The same scanner can also be configured differently. Frequent software updates might have an influence on images produced. A major consequence of these scanner and protocol variations is a domain shift [22], i.e., a shift in data distribution across various centers/time/machines/software. Please see Figure 1 showing inter-center variation in data distribution obtained from PET/CT scans from HEad and neCK TumOR (HECKTOR) challenge [23].

Studies have shown the effects of image acquisition parameters on the reproducibility of radiomic features [21,24,25,26,27]. Many studies [1,28,29,30,31] have also explored the discriminative power of radiomic features. However, the reproducibility of a radiomic feature does not guarantee its discriminative power [32,33], and thus the two aspects of reproducibility and discriminative power cannot be treated in isolation. For instance, a feature may have excellent reproducibility across scanner and protocol variations but have no discriminative power for the problem of interest. The scanner and/or protocol variability could hamper the stability as well as the discriminative power of the features. Feature variability is also caused due to varying contours or ROIs. For example, Yang et al. [34] observed that gray-level neighborhood difference matrices (GLNDM) based radiomic features were most robust against the manual contouring variability in PET scans of lung cancer. Variation in inter-observer delineation has an impact on radiomic analysis and is examined in [34,35,36,37,38]. These variations can have repercussions on image texture and consequently on the radiomic features. Different feature extraction algorithms and image processing techniques also influence the feature variation and have been addressed by the image biomarker standardization initiative [39]. However, in this work, we only focus on studies that investigate radiomic feature reproducibility across scanner and protocol variations. Various methods have been proposed in the literature to improve the reproducibility of radiomic features across scanner and protocol variations and a few of these harmonization methods have been reviewed in [40,41]. In addition to feature robustness, investigations should be carried out to ascertain model accuracy/performance as well. For instance, the model should have sufficient data to achieve predictive performance at least equal to the current clinical standard; the model should be externally and/or internally validated across different centers; several performance metrics such as the area under curve (AUC) of the receiver operating characteristic (ROC) curve and precision recall (PR) curves can be used to evaluate the model performance.

**Figure 2:**
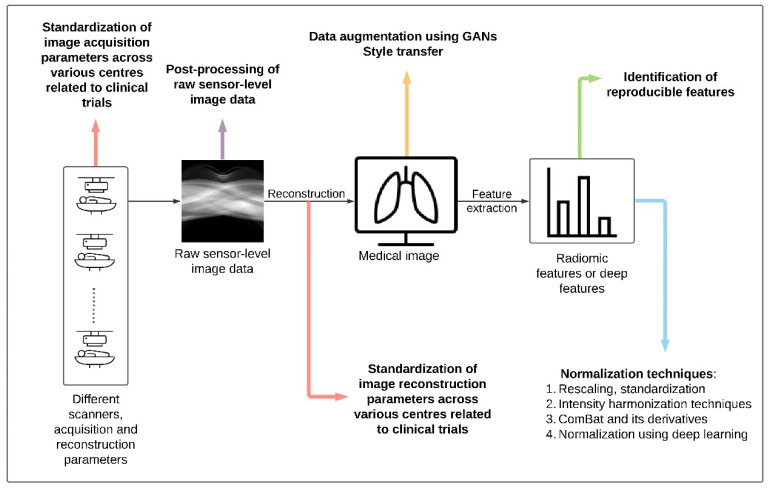
Overview of harmonization methods at different stages of medical imaging.

The organization of the paper is as follows. We primarily categorize the methods under the image domain or the feature domain. The harmonization methods discussed under the image domain (Section 3) are performed on the whole image (raw or reconstructed) before feature extraction and thus aim to harmonize images acquired across different centers/scanners/protocols. In this section, we briefly review methods in such a way that they can be applied at every stage of medical image processing from image acquisition to image analysis (Figure 2). This section starts with a discussion on various standards for image acquisition/reconstruction parameters. Moving forward, post-processing methods for raw sensor-level image data followed by brief reviews of existing image analysis techniques (e.g., data augmentation techniques using generative adversarial networks (GANs) and style transfer) are discussed. The methods categorized under the feature domain (Section 4) are performed after (or within) feature extraction and aim to harmonize extracted radiomic features. In this section, the methods are listed in order of their complexity. Under the feature domain, we briefly review two approaches: identification of reproducible features (a convenient approach) and normalization techniques (statistical approaches). The normalization techniques are further divided into basic statistical normalization (rescaling/standardization); intensity harmonization techniques; ComBat method and its derivatives; normalization using DL. The overall objective of this review is to address the advantages, disadvantages, and challenges posed by these harmonization methods. Figure 2 shows an overview of different harmonization methods that are applicable at different stages of medical imaging.

Search strategy: Our search strategy for this review was based on a set of research questions:

1. Have scanner and protocol variations affected the reproducibility of radiomic features/images? If yes, the how significant was the change?
2. Various harmonization methods were identified in previous work. Can they be categorized into domains (image and feature)? Furthermore, can the methods be applied at different stages of medical imaging (Figure 2)?
3. What are the latest developments in the field of radiomics to make radiomics more reproducible?
4. Are there non-medical studies performed to harmonize images/features? What are the different types of methods?
5. What are the advantages, disadvantages and challenges of various harmonization methods?

Keeping in mind the above research questions, we searched for literature using PubMed and Google Scholar by typing in the following keywords: “radiomics”, “harmonization methods”, “feature reproducibility”, “robustness”, “scanner variation”, “protocol variation”, “deep learning”, “multicentric studies”, “medical imaging”. Articles were selected based on their novelty, relevance, and being in English. 

## 2. Image Domain Harmonization

### 2.1. Standardization of Image Acquisition and Reconstruction Parameters across Various Centers Related to Clinical Trials

For multicentric prospective studies, the ideal way to standardize radiomic features is to define and follow imaging protocols that define scanner types in conjunction with acquisition and reconstruction parameters (see Table 1 for summary). For example, the European Society for Therapeutic Radiology and Oncology (ESTRO) panel provides guidelines for procedures and methods for image-guided radiation therapy (IGRT) in prostate cancer [42,43]. This panel consulted a large base of the radiation oncology community from the European Union and developed guidelines for delineating localized prostate cancer in CT and magnetic resonance images (MRI). ESTRO also has a working group focusing on cervical carcinoma for developing and validating methods and imaging parameters from various institutions [44]. For standardization of PET imaging, the European Association of Nuclear Medicine (EANM) [45] launched the EARL (EANM Research Ltd.) program covering areas such as scan acquisition, processing of images, and image interpretation. Pfaehler et al. [46] conducted a study to investigate the effects of harmonizing image reconstructions on feature reproducibility and concluded that EARL compliant image reconstruction harmonized a wide selection of radiomic features. A similar initiative by the American Society for Radiation Oncology (ASTRO) [47] was created to develop a ‘practice parameter’, for IGRT and to provide quality assurance standards, personnel qualifications, indications, and guided documentation [48] for imaging. In MRI, however, such guidelines do not exist [49] and most of the MRI modalities are not even quantitative [50]. Efforts have been taken in the past, concerning MRI imaging, for example by UCHealth [51] to reduce the number of MRI protocols from 168 to 66 across scanners and centers by selecting an appropriate clinics-driven protocol and standardization process. Another set of guidelines is provided by the FDA (Food and Drug Administration) [52] to focus on image acquisition in clinical trials conducted to support the authorization of drugs and biological products. Ever since this draft by FDA was released in 2015, it has become a reference standard for most promoters and industries of clinical trials.

Such efforts need to be extended to the radiomics field to help control the variability present across different scanner machines, acquisition and reconstruction parameters. However, these radiomics guidelines might not be able to account for the plethora of existing scanners, protocols, and reconstruction parameters by different vendors across multiple centers.

### 2.2. Post-Processing of Raw Sensor-Level Image Data

It would be worthwhile to work with raw sensor-level data, right before reconstructing the image and apply harmonization methods on it to remove scanner and protocol variability. Image reconstruction, necessary for human viewing and interpretation, combined with the manual contouring variability, could lead to a loss of latent raw sensor-level image data and lower precision in measurements. Most machine learning (ML) and DL algorithms have been used on reconstructed images in the existing medical imaging workflow. Instead, the abilities of ML and DL could be leveraged to process the underlying raw sensor-level data to access its hidden nuances [53,54,55]. A study conducted by Lee et al. [56] investigated the performance of a CNN for classifying raw CT data in the sinogram-space to identify the body region and detect intracranial hemorrhage. The sinogram-specific CNN performed slightly better than the conventional neural network (Inception-V3 [57]) in the image-space by approximately 3% in terms of accuracy. In another study, Gallardo-Estrella et al. [58] proposed a method to reduce variability due to different reconstruction kernels in CT images by decomposing each CT scan into a set of frequency bands and the energy in each frequency band is scaled to a reference value iteratively. This method was validated for emphysema reconstruction. Although this method was applied to normalize fully reconstructed images, the applicability of this method could be extended to harmonize raw image data. Radiomics signature analysis can also be performed directly on the raw image data without the need for reconstruction which adds bias and variability [56,59]. Furthermore, the reconstruction process itself can also be considered as a prediction problem utilizing raw CT data (sinograms) or k-space values of MRI inputs [60]. These studies widen the scope to apply harmonization methods on raw image data and take advantage of the hidden information in the raw image data rather than applying it in the reconstructed image-space. Refer to Table 2 for a summary of this section.

### 2.3. Data Augmentation Using GANs

ML-based techniques have emerged to provide effective solutions to translate images across various domains by harmonizing images as opposed to radiomic features alone. Examples include ML-based adaptive dictionary learning [61] and DL methods like using GANs [62,63,64,65,66,67,68,69,70]. Methods using coefficients of spherical harmonics to harmonize diffusion MRI have been explored [61,71,72,73]. The applicability of this method was limited to diffusion MRI since the analysis of diffusion MRI requires various processing steps to correct for scanner acquisitions and protocol variation effects and was addressed by the 2018 CDMRI (computational diffusion MRI) Harmonization challenge [74].

Another widely used DL technique in medical image analysis are GANs [75] because of their ability to model target data distributions to generate realistic images (summary in Table 3 at the end of this section). GANs consist of two adversarial networks, a generator that generates realistic data and a discriminator that distinguishes whether the data is real or fake. The objective of a GAN is to keep the generator and discriminator in opposition to each other. Despite the difficulty in handling multi-centric medical data, GANs have shown promising results to overcome the multi-center variation. Zhong et al. [76] used a dual GAN, with U-Net [77] as the backbone, to harmonize the diffusion tensor imaging (DTI) derived metrics on neonatal brains and compared it with three other methods: voxel-wise scaling, global-wise scaling, and ComBat. The results from this study showed that the GAN based method performed better at harmonizing neonatal datasets in multi-centric studies. Another study by Modanwal et al. [78] used a cycleGAN [64] to perform intensity harmonization on MRI breast images obtained from two scanners (GE and Siemens). A cycleGAN utilizes a cycle consistency loss to translate an image from one domain to another without the requirement for paired data. Cycle consistency loss is an optimization problem in the sense that if a zebra image is converted to a horse image and back to being a zebra image, we should obtain the same input in return. This method was adapted by modifying the discriminator that further helped in preserving the tissue characteristics and shape. This method could operate on unpaired images; however, a downside is that this algorithm worked only for 2D slices and could not retain volume information due to limited computational resources. A comparative study was conducted by Cackowski et al. [79] between ComBat and cycleGAN to harmonize multi-centric MRI images. The authors found that both methods were complementary to each other and had similar effects on the radiomic features. The grey-level run length matrix (GLRLM) features benefited more from ComBat while the cycleGAN performed better on Gray Level Size Zone (GLSZM) features. It would be of great interest to see the effects the combination of ComBat and GAN would have on radiomic features.

**Figure 3:**
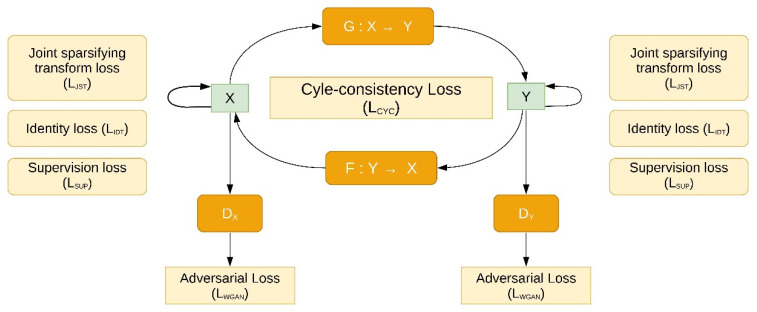
Basic GAN-CIRCLE network. Here, X is a set of LR CT scans and Y is the corresponding HR CT scans. The network has two GAN modules, a low-to-high image reconstructor (Generator G, Discriminator DY) and a high-to-low image reconstructor (Generator F, Discriminator DX). Different loss functions are harmoniously coupled for training the network and monitored with regularized cycle-consistency and identity loss to prevent overfitting. Figure is adapted from [63].

Guha et al. [63] conducted a study that transforms low-resolution (LR) CT scans of trabecular (Tb) bone microstructures into high-resolution (HR) CT scans, obtained from two scanners (LR from Siemens FLASH and HR from Siemens FORCE; paired images), using GAN-CIRCLE, of which the architecture is shown in Figure 3. This DL-based method was inspired by You et al. [80] and is monitored by three losses: the identical, residual, and cycle consistency loss. The cycle consistency establishes an end-to-end nonlinear mapping from LR CT to HR CT scans with reference to the Wasserstein distance [81]. This type of loss was first used in cycleGANs [64] and it helps a GAN to perform image-to-image translation between unpaired images by enforcing a strong consistency across domains. The residual network is built to preserve the high frequency anatomical details in the image. The identity loss aids to regularize training by learning sufficient latent structural information to enhance the image resolution. The results were compared to and evaluated against the reference value obtained from the true HR CT scans. The predicted results showed improvement in the structural similarity index with respect to true HR CT scans in terms of Tb network area density, Tb thickness and Tb spacing. Other authors [82,83,84,85,86] have also addressed image up-sampling using DL techniques.

**Figure 4:**
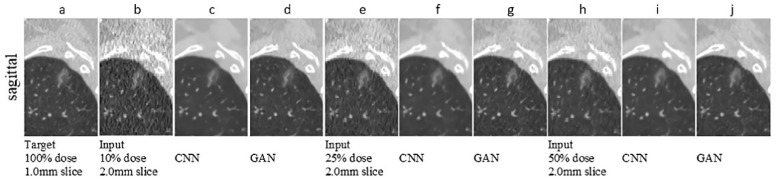
Normalization results reused from [87] with original copy obtained from authors. The row represents a sagittal view of the scans containing the ROI nodule. Column (**a**) represents the target image; Column (**b**,**e**,**h**) shows the input images with different slice thicknesses and dosage; Columns (**c**,**f**,**i**) show the CNN results and Columns (**d**,**g**,**j**) show the GAN-based results.

A similar study to [63] was conducted by Wei et al. [87] who used a 3D GAN to normalize CT images obtained from different slice thickness and dosage scenarios. This method used a unique spectral-norm layer inspired by Miyato et al. [88] to perform global regularization in the feature matrices. The GAN-based approach resulted in an improved perceptual appearance and reduced the variability across radiomic features. This method is unique, since it performs de-noising and super-resolution simultaneously. However, no clinical study (e.g., diagnostic task) was performed using the normalized images to check its feasibility. Figure 4 shows the normalization results obtained by the authors [87].

Despite their novel design, training GANs can be challenging due to disappearing gradients. While back propagating the loss, the gradient flows backwards from the final layer to the first layer. The gradient gets extremely small, slowing down the learning process in the initial layers or even stopping completely. This halts the training of initial layers because the gradient does not change the weights anymore. GANs are also prone to generate images with similar appearance as an effect of mode collapse [89] which occurs when the generator produces only a limited or a single type of output to fool the discriminator. Due to this the discriminator does not learn to come out of this trap resulting in a GAN failure. Apart from this, GAN-based models can also add unrealistic artefacts in the images [87].

### 2.4. Style Transfer

The advances in the field of style transfer may prove useful to overcome scanner acquisition and reconstruction parameter variability at the image level. Style transfer is a computer vision technique that requires two images, a content image and a reference style image, and combines them so that the resulting output image preserves the key elements of the content image but appears to be “painted” in the style of the reference style image. When there is no radiomics model available for a new scanner or protocol, style transfer could be applied such that the images coming from a new machine can be transformed so that they look like they were acquired from an existing machine [90]. This section discusses various style transfer methods (Table 4 at the end of this section), starting with the non-CNN methods followed by neural style transfer (NST) methods. We categorize and briefly explain the existing neural style transfer methods and discuss their strengths and weaknesses.

Before the onset of neural style transfer, image stylization came under the category of non-photorealistic rendering (NPR). Image-based artistic rendering (IB-AR) [91,92,93,94] is the artistic stylization of two-dimensional images and can be further categorized into four categories; stroke-based, region-based, example-based, and image processing and filtering. [94]. Stroke-based rendering tries to render strokes (e.g., tiles, stipples or brush strokes) on a content image to adapt to a particular style [95]. However, this method is built to adapt to only one particular style and not arbitrary styles [94]. Region-based rendering [96,97] renders stroke patterns in semantic regions of an image and even though it permits local control over the degree of details, this method also cannot be adapted for arbitrary styles [94]. Hertzmann et al. [98] proposed ‘image analogies’ to learn the mapping between paired source and target images in a supervised fashion but paired images are often not available in practical settings. Even though filtering and image pre-processing [99,100] are efficient and straightforward techniques, they might not be entirely applicable to a wide variety of styles [94]. The above-mentioned techniques do provide dependable stylized results, but their limitations eventually gave rise to novel methods in the field of NST.

**Figure 5:**
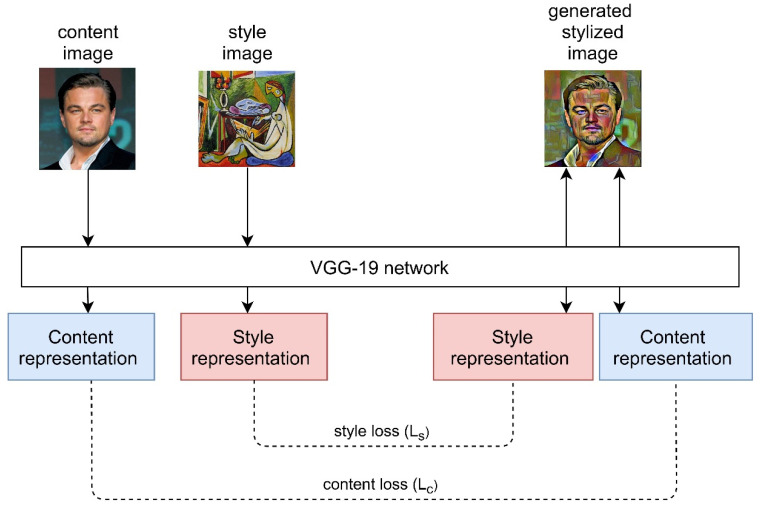
Illustration of concept of neural style transfer using original work [101].

The groundbreaking work of Gatys et al. [101] paved the way for a new field of NST. Gatys et al. [101] first conducted a study that separates content from one image and style from another image and combines it into a new image using a neural network (Figure 5). The paper demonstrated that transferring style from one image to the other can be modelled as an optimization problem that can further be solved by training a neural network, VGG-19 [102] in this case. The style was extracted by looking at the spatial correlation between filter responses and this was calculated as the Gram matrix [103] of a feature map. The total loss was calculated as the weighted sum of both content loss (Lc) and style loss (Ls) by weights α and β respectively. Thus, the style transfer task was reduced to creating a new image through an optimization process by minimizing the total loss. However, the high resolution of images affected the speed of the style transfer process and the algorithm failed to preserve the consistency of details and fine structures during style transfer because the low-level information was not retained by the CNN. The Gram matrix is not the only choice for representing style in images. There are also other interpretations of Gram matrix, such as MMD mathematically proven by Li et al. [104]. Additionally, the definitions of style and content remain unclear since no representation exists to factorize either style or content of an image. 

Li et al. [104] questioned the usage of the Gram matrix from Gatys et al. [101] and were not satisfied with the motivation behind its use. They treated neural style transfer as a domain adaptation problem where the difference between the source distribution and the target distribution would be measured and minimized. They provided mathematical proof that matching Gram matrices of filter responses is equivalent to minimizing MMD [105] with the second-order polynomial kernel. The VGG19 network was used here as well, and they proved that the top layers had larger receptive fields and could reproduce more global textures.

Xu et al. [106] proposed a method for arbitrary style transfer, which allows the stylization of images from an unseen content image and style image. They utilized the Behance dataset [107] containing several artistic images and course category labels for style and content. They combined the concepts of original neural style transfer with the concept of adversarial training for arbitrary style transfer from multi-domain images. Xu et al. [106] built a conditional generator to fool the discriminator and to assure that the style and content representations are similar to the input images by combining content and style using adaptive instance normalization (AdaIN) [108]. The method utilized Gram loss for the style representation, perceptual loss [109] for content representation and adversarial loss to capture beyond texture the style information from a distinct style label/category. Their methods outperform previous work using AdaIN [108] and whitening and color transform [110] quantitatively. However, qualitative results in this study show that stylization does not occur beyond a point even after tuning the parameters due to the difficulty of the optimization.

A medically relevant study led by Yang et al. [111] investigated the effects of different kernels on CT images and proposed an unsupervised kernel conversion method by utilizing a cycleGAN with AdaIN [108] that works on unpaired images. They modified the base model of UNet [77] to use polyphase decomposition [112] which resulted in better performance. They assumed that the unsupervised kernel conversion problem can be posed as an unsupervised image style transfer problem that can be solved using optimal transport [113,114]. The qualitative results showed that their methods performed better however, in the quantitative evaluation (peak signal to noise ratio and structure similarity index), supervised learning performed better than unsupervised learning. A similar study by Liu et al. [115] was carried out to harmonize MRI images from multiple arbitrary sites using a style transferable GAN. They treated harmonization as a style transfer problem and proved that their model applied to unseen images provided there was enough data available from multiple sites for training purposes. However, the model only worked on two-dimensional images and not three-dimensional images. They also mention that selecting an appropriate reference image would be challenging if the data pool was vast.

**Figure 6:**
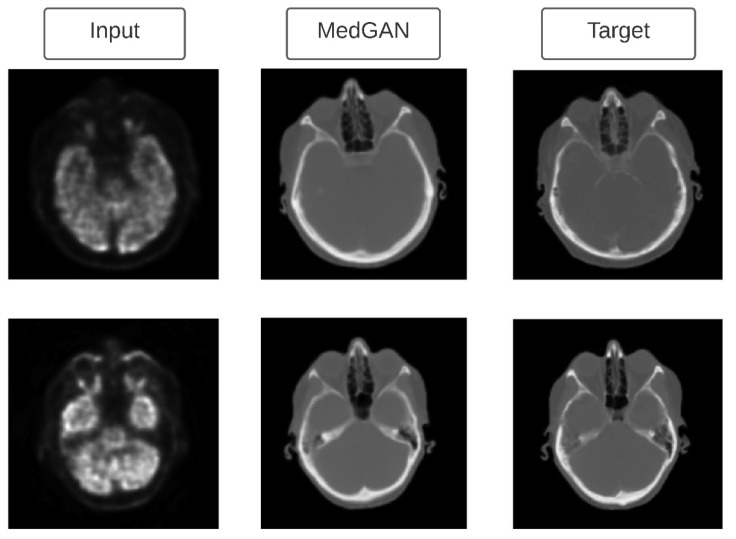
PET to CT translation using MedGAN. Figure adapted from [116] with permission from Elsevier.

Studies by Armanious et al. [116] and Clancy and Milanko [117] have also utilized the concept of style transfer to perform image-to-image translation between PET-CT images (see Figure 6) and healthy-unhealthy chest X-rays respectively. The difference between both the studies is that Armanious et al. [116] utilized style transfer losses [101] to match the texture between the stylized image and the target image, while Clancy and Milanko [117] just used the cycleGAN and adversarial losses to perform style transfer. Moreover, the MedGAN created by Armanious et al. [116]) incorporates a novel generator CasNet, which is a cascade of UNet blocks to obtain sharper translated images. MedGAN seemed to outperform other existing image-to-image translation methods (e.g., pix2pix [118] and perceptual adversarial network [119]) by providing quantitative and perceptual assessments. Another study by Fetty et al. [120] investigated how the latent space can be manipulated to obtain high-resolution scans by utilizing their StyleGAN architecture. Their StyleGAN architecture incorporated AdaIN [108] method for transferring style. StyleGAN was trained on MRI to CT images (with pelvic malignancies) and achieved a root mean squared error of 0.34 for CT-MRI translation and a mean absolute error of 59 HU for MRI-CT translation.

Many more such studies [121,122,123,124,125,126] were conducted by applying style transfer methods on medical images, and this approach has the potential to harmonize images, either by image-to-image translations or domain transformations. Depending on the architectures used to perform style transfer, paired or unpaired images might be needed, e.g., if harmonization is to be performed using cycleGAN or StyleGAN as a baseline then paired images are not a requirement. Losses can be modified in such a way that they may or may not include style and content losses from Gatys et al.’s [101] method. In case GANs were to be used for NST, the developer should be mindful of limitations that GANs pose, as discussed in the previous section.

## 3. Feature Domain Harmonization

### 3.1. Focusing on Reproducible Features (Identification of Reproducible Features)

These studies test the reproducibility, variability, and repeatability of features extracted from various phantom and patient studies over different reconstruction and acquisition parameters in the case of multi-centric datasets and examine the reproducibility of radiomic features. Refer to Table 5 for a summary.

In the context of PET images, a study by Shiri et al. [127] investigated the impact of various image reconstruction settings on several PET/CT radiomic features obtained from a phantom dataset (developed in-house National Electrical Manufacturers Association [NEMA]) and a patient dataset from two different scanners. Radiomic features were grouped into intensity-based, geometry-based and texture-based features and their reproducibility and variability were evaluated using the coefficient of variation (COV). The results from both phantom and patient studies showed that 47% of all radiomic features were reproducible. Almost half of intensity-based and texture-based and all the geometry-based features were found to be reproducible respectively. The intensity and geometry-based features were also found to be reproducible in another study by Vuong et al. [128], where the authors investigate if the PET/CT radiomics models can be transferred to PET/MRI models by checking the reproducibility of radiomic features against different test-retest and attenuation correction variability. However, Shiri et al. [127] used a phantom body filled with homogeneous activity rather than heterogeneous activity, which does not properly imitate the human tissue. The respiratory motion [127,128], quantization [127,128] and segmentation parameters [127] were also absent in the studies, which may have had a considerable effect on the radiomic features. A similar study by Bailly et al. [129] analyzed the reproducibility of texture features in PET scans across different acquisition and reconstruction parameters in the context of multi-center trials. They found out that only a few features were strongly reproducible and acceptable for multi-center trials. Nevertheless, this study checked the reproducibility of texture features evaluated against reconstruction parameters coming from just one manufacturer. Many such studies have been carried out to check the reproducibility of radiomic features in PET scans [130,131,132,133,134,135,136,137,138,139,140,141,142,143,144] but most of them only check the impact of variability in scanner and imaging parameters and do not provide concrete image and/or feature harmonization methods to obtain reproducible features.

In the case of CT scans, Prayer et al. [145] conducted a trial to investigate the inter-and intra-scanner repeatability and reproducibility of computed tomography (CT) radiomic features (radiomic feature) of fibrosing interstitial lung disease (fILD). The dataset was obtained from IRB-approved test-retest study with sixty fILD patients. The results showed that intra and inter-scanner reproducibility were highly affected by the variation in slice thicknesses than the variation in reconstruction kernels under study and were reconstruction parameter-specific respectively. The CT radiomic features showed excellent reconstruction parameter-specific repeatability for the test-retest study. However, the sample size of the data used was small, and to check the variability of features only two scanners were used. Careful selection of radiomic features is critical to ensure plausible outcomes in heterogeneous CT datasets. Similar studies have been conducted in the past where the reproducibility of CT radiomic features was investigated using phantom data [25,26,27,146] as well as patient data [20,147,148]. The phantom studies were carried forward to reduce the exposure to patients however, they are not real substitutes of heterogeneous human tissues.

Considering MRI, a recent study using radiomics to investigate the reproducibility of features across several MRI scanners and scanning protocol parameters was carried out using both phantom data and patient (volunteer) data by Lee et al. [149]. This study also investigated the repeatability by measuring the variability of radiomic features using a test-retest strategy. The variability of radiomic features across different MRI scanners and protocols was evaluated using the intra-class correlation coefficient (ICC) and the repeatability was evaluated using the coefficient of variation (COV). The COV measurements showed that there was very little difference in the variability between filtering and normalizing effects which were used for pre-processing. The ICC measurements showed higher repeatability for the phantom data than for the patient data. However, this study was not able to prevent the effects of the volunteer’s movements on the radiomic values despite simulating movements while scanning. A similar study, conducted by Peerlings et al. [150], extracted stable parametric MRI radiomic features with a minimum concordance correlation coefficient of 0.85 between data derived from 61 patients’ test and retest apparent diffusion coefficient (ADC) maps across various MRI-systems, tissues and vendors. A review by Traverso et al. [151] mentions that there are not many phantom studies conducted to investigate the reproducibility of MRI radiomic features. Most of them cover various sites such as the brain [152,153], the gastro-intestinal tract [154,155,156] and the prostate [157,158], although this limitation was addressed by Rai et al. [159] by developing a novel 3D MRI radiomic phantom to assess the robustness and reproducibility of MRI radiomic features across multiple centers.

### 3.2. Normalization Techniques

Many statistical normalization methods have been proposed in the past and have calculated the benefits of applying normalization techniques for harmonizing radiomic features affected by variability in scanner acquisition protocols and reconstruction settings.

#### 3.2.1. Statistical Normalization

Chatterjee et al. [160] investigated the effect of applying rescaling and standardization (zero mean, unit standard deviation) as normalization transformations in MRI images obtained from two different institutes with outcome as lymphovascular space invasion and cancer staging. These transformations were applied separately on balanced training and testing sets rather than applying normalization for the entire dataset. This method enhanced the predictive power of the radiomic models through external validation from an external institute. The average prediction accuracy of radiomic features increased from 0.64 to 0.72, average Matthews correlation coefficient (MCC) increased from 0.34 to 0.44 and average F-score increased from 0.48 to 0.71. A similar study by Haga et al. [161] used z-score normalization to standardize the radiomic features extracted from CT images of NSCLC (non-small cell lung cancer) patients from The University of Tokyo Hospital and TCIA (the Cancer Imaging Archive). Z-score normalization uses the formula:

(1) $$z=\frac{\left(x-\bar{x}\right)}{s}$$

where x is the feature, $\bar{x}$ is the mean and s is the standard deviation and this method gave the best prediction radiomic model with 0.789 AUC (area under the receiver observed characteristics curve) when compared to min-max normalization (0.725 AUC) and whitening from the principle component analysis (0.785 AUC). Refer to Table 6 for a summary.

#### 3.2.2. Intensity Harmonization Techniques

Crombé et al. [162] performed intensity harmonization techniques (IHT) as a post-processing method on T2-weighted MRI images of sarcoma patients to enhance the MFS (metastatic-relapse-free survival) predictive models. They compared standard normalization, z-score normalization, standardization per signal intensities of healthy tissue, histogram matching and ComBat harmonization methods. A histogram is a statistical representation of an image, which shows the distribution of intensity values. It does not contain information about the location of the image pixels. Histogram matching is where intensity histograms are aligned to a reference intensity histogram. In this study, intensity histogram matching performed better with an AUC of 0.823 in an unsupervised analysis. Related studies [163,164,165] have used histogram matching to normalize MRI intensity scales. A few studies [166,167] have also applied histogram equalization (enhancing the contrast by flattening the histogram) on images to normalize intensity scales to pre-process images before applying a ComBat harmonization method on top of it. Refer to Table 7 for summary.

#### 3.2.3. ComBat Method and Its Derivatives

ComBat harmonization is a statistical method that was developed originally to harmonize gene expression arrays [168]. ComBat was designed to provide estimates of the effects of assigned batches -which have a single technical difference between each other, while taking into account the effect of biological covariates on the variables or features being harmonized. The estimations are calculated using Bayesian models, and a location/scale shift is performed accordingly to adjust the values of different features. The application of ComBat on radiomic features was first introduced by Fortin et al. [169]. The authors used ComBat to harmonize cortical thickness measurements calculated on diffusion imaging tensor data to remove variations in feature values attributed to differences in acquisition and reconstruction parameters. The authors reported that ComBat removes interscanner variability for these measurements and can also preserve biological correlations. The authors further developed an open software for ComBat that can be used for radiomics analysis.

**Figure 7:**
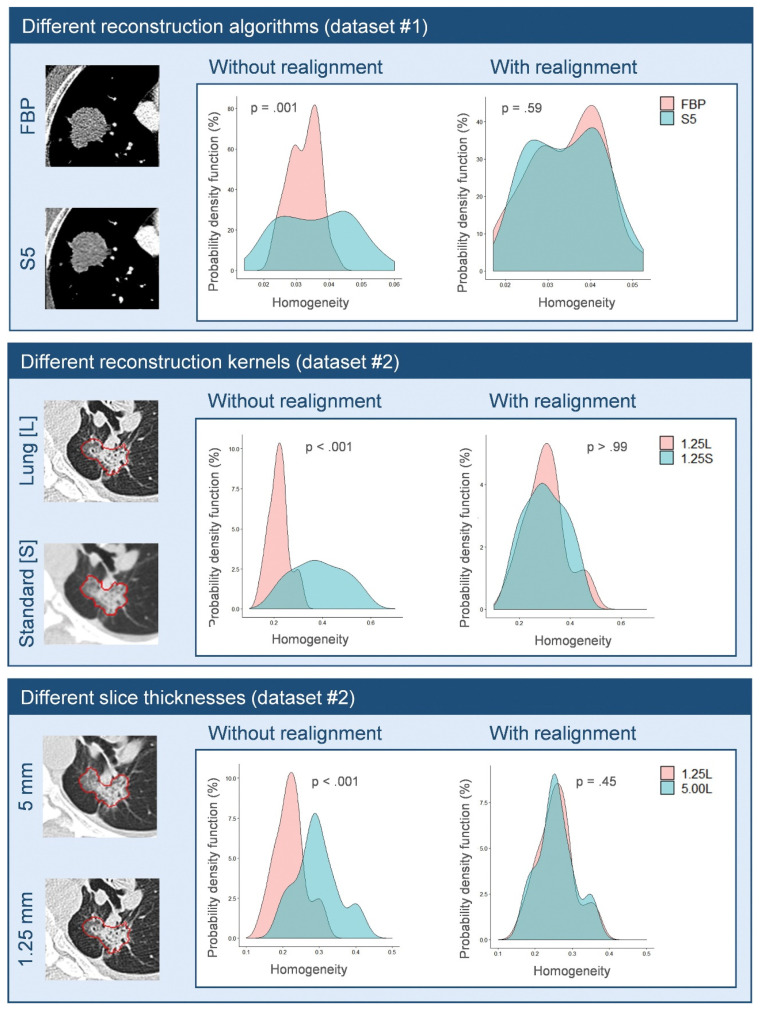
Probability density function of homogeneity before and after applying ComBat for realignment between different CT reconstruction algorithms, reconstruction kernels and slice thicknesses. FBP: filtered back-projection. Figure reproduced from [170]. Figure reproduced with copyright permission from The Radiological Society of North America.

Following that, several studies further investigated the potential of ComBat harmonization in radiomics analyses. Orlhac et al. [170] investigated the potential of ComBat to correct for the variations of CT radiomic features extracted from scans collected from different centers. The authors reported that all radiomic features were significantly affected by differences in acquisition and reconstruction parameters, and that almost all radiomic features can be used following ComBat harmonization. The authors further reported an improvement in the performance metrics of the developed radiomic signatures after ComBat harmonization. Figure 7 shows the result for this study [170] with three instances of feature distributions realigned between different CT reconstruction algorithms, reconstruction kernels and slice thicknesses. Another study by Orlhac et al. [171] investigated the potential of ComBat to harmonize radiomic features extracted from PET scans acquired differently. The authors reported similar results to that of the application of ComBat on CT scans. A similar study investigated the performance of ComBat harmonization, in addition to modified ComBat methods: M-ComBat, B-ComBat, and BM-ComBat [172]. The study reported a significant improvement in the performance of radiomic signatures following the application of all the investigated ComBat methods.

Of note, none of the above studies investigated the concordance (reproducibility) of features after ComBat harmonization. Data with similar distributions could still have different individual data points within. Furthermore, the aim of radiomics is to improve personalized medicine. Therefore, for clinical applications, the radiomic signature is expected to be applied on a single patient each time, and not a group of patients simultaneously. Henceforth, the focus of harmonization techniques must be the standardization of radiomic feature values across different imaging settings and patient populations. This is statistically translated into the assessment of concordance in features values following harmonization, and not the performance of developed signatures following harmonization [173].

With regards to the application of ComBat on radiomic features, several points must be taken into consideration: (i) In contrast to gene expression, radiomic features have different complexity levels. Therefore, ComBat is not expected to perform uniformly on all features; (ii) Biological covariates are embedded in the harmonization equation, and as the aim of radiomic studies is to investigate such relationships, biological covariates cannot be provided for the ComBat formula. Furthermore, as the reproducibility of a feature is a cornerstone for it to be further analyzed, solely harmonizing the distribution without paying attention to individual value and rank, is not expected to be beneficial for the generalizability of radiomics signatures. Therefore, the concordance in feature values following ComBat harmonization must be used as an initial feature selection step, to select features that become concordant for further analysis. A framework that guides the use of ComBat in radiomics analyses was published [174]. This framework consists of several steps. The first step is to collect the imaging dataset(s), and to extract the imaging acquisition and reconstruction parameters. Following this, an anthropomorphic phantom is scanned with the different acquisition and reconstruction parameters used for acquiring the scans in the patients’ imaging dataset. Radiomic features are then extracted from the phantom scans, and the reproducibility of radiomic features is assessed on those scans using the concordance correlation coefficient (CCC) [175], and the reproducible features (CCC > 0.9) could be further used for further modeling. To assess the performance of ComBat, it is applied on the phantom scans, followed by the calculation of the CCC. Radiomic features that obtain a CCC > 0.9 following ComBat application are to be considered “ComBatable”.

One study applied the framework on thirteen scans of a phantom [176] acquired using different imaging protocols and vendors. The study investigated the reproducibility of radiomic features in a pairwise manner, resulting in a total of seventy-eight pairs. The study reported that different numbers of reproducible radiomic features were identified in each scenario. The results confirmed that radiomic features are affected differently by the differences in imaging protocols and vendors used, with a wide range between nine and seventy-eight reproducible features, substantiating the need for the application of the framework for all radiomic studies [177]. The study also reported that ComBat harmonization did not perform uniformly on radiomic features, and the number of features that could be used following ComBat harmonization ranged between fourteen and eighty radiomic features. Henceforth, the study recommended that the application of ComBat harmonization should follow a similar impact analysis depending on the data under analysis.

Another study utilized a similar framework to assess the performance of ComBat on CT phantom scans that were acquired with the same acquisition and reconstruction parameters except for the in-plane resolution [178,179], on two different scanner models. The authors performed pairwise comparisons between the scans and reported that radiomic features are affected differently by the degree of variation within a single reconstruction parameter (in-plane resolution). A given radiomic feature can be reproducible up to a certain degree of variation in pixel spacing but becomes unreproducible when the variation is relatively large. Other features were found to be reproducible regardless of the variation in pixel spacing, while a few features were found to vary significantly with the slightest change in pixel spacing. These groups of features differed based on the scanner model used to obtain the scans. The application of ComBat on those scans resulted in a different number of reproducible features depending on the variation in the scan in-plane resolution, which also varied according to the scanner model. As such, the study recommended the assessment of the reproducibility and the harmonizability (using any harmonization method) of radiomic features in the data under study before performing radiomics analyses. Refer to Table 8 for summary of ComBat method and its derivatives.

#### 3.2.4. Normalization Using Deep Learning

**Figure 8:**
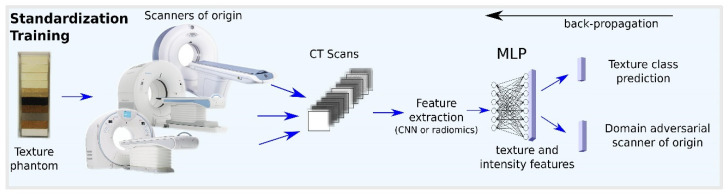
An overview of the proposed normalization method by [21] using a CT phantom. Figure adapted from [21].

Andrearczyk et al. [21] proposed a DL-based technique trained on phantom data to normalize various types of features including hand-crafted and deep features. The main idea is to use a simple neural network (two layers in [21]) to learn a non-linear normalization transformation. This work is based on the assumption that training a deep model on top of features to classify texture types while being adversarial to the scanner of origin creates features that are stable to scanner variations. It therefore aims at reducing intra-scan clustering that does not underline true physio-pathological tissue changes, while maintaining highly informative and discriminative features. The generalization of the proposed approach to unseen textures and unseen scanners is demonstrated by a set of experiments using a publicly available CT texture phantom dataset scanned with various imaging devices and parameters. It is assessed by training the model on a subset of classes and scanners and evaluating the stability on the remaining ones. The stability of the normalized features is demonstrated by the increased ICC, clustering based measures showing the class separability, as well as reduced correlation with pixel spacings. The phantom used for this method was developed in [27]. It contains 10 cartridges of different textures and was scanned by 17 different scanners and acquisition settings. Refer to Figure 8 for an overview of their proposed method. Using a phantom allows a controlled analysis that isolates the variation due to scanner variation from other variations related with patient acquisition. Phantoms can also be scanned by specific scanners with special clinical settings to specifically improve the normalization of the features for clinical use. The normalization could therefore be updated to follow the latest imaging advances and standards. However, while this phantom was designed to mimic actual biomedical tissue types (particularly non-small cell lung cancer), the method has yet to be validated on real patient data.

Studies by Rozantsev et al. [181] and Sun and Saenko [182] have adapted divergence-based approaches for domain adaptation by using a two-stream CNN architecture (one in the source domain with synthetic images and the other in the target domain with real images) with unshared weights and the DeepCORAL [183] architecture, respectively. Their methodologies provided a domain-invariant representation by trying to reduce the divergence (reduce the gap/distance) between feature distributions of source and target data distributions (both use non-medical images). Rozantsev et al. [181] used maximum mean discrepancy (MMD) to determine if two samples have the same distribution and Sun and Saenko [182] used correlation alignment that attempts to align the second-order statistics of two distributions by applying a linear transformation. [181] obtains an average accuracy of 0.908 while [182] got an average accuracy of 0.72, both using the Office dataset [184]. However, if these methods were to be applied to medical images, the assumption that scanner information can be eliminated by a simple definable constraint could probably work for linear systems like CT rather than for complex nonlinear systems such as MRI. To make domain adaptation techniques widely applicable, domain adversarial neural networks (DANNs) [185,186] have been explored to increase the invariance of the transformed features to the scanner of origin. DANNs use a label predictor and a domain classifier to optimize the features to make the learned features discriminative for the main task but non-discriminative between the domains. Adapting the same framework as proposed in [186], Dinsdale et al. [187] utilized an iterative update approach that aimed to generate scanner-invariant (i.e., harmonized features) representations of MRI neuroimages while evaluating the main task (segmentation), thus decreasing the influence of scanner variation on the predictions. Refer to Table 9 for a summary of normalization methods using DL techniques.

## 4. Discussion

With the emergence of Radiomics within medical image analysis comes the challenges associated with it which could hamper the growth of the field. Both methodologies, traditional hand-crafted features and DL, are faced with standardization issues. The hand-crafted features are most of the time not standardized when the data under analysis is acquired with different scanner acquisition protocols and/or reconstruction settings and there is also a lack of biological correlation of these features. To overcome these limitations, various standardization/harmonization techniques have been introduced and utilized.

In the image domain, the methods mentioned above are applicable on images (raw or reconstructed image). Certain regulations and guidelines can be implemented in imaging protocols by providing quality assurance, indications and guided documentation such as the one laid down by ESTRO and FDA. Such guidelines are not available extensively for MRI [49] but efforts have been taken to reduce the number of MRI protocols [51]. However, these guidelines might not be able to compensate for the number of existing scanners and protocol combinations. Apart from setting guidelines, models can be developed using ML and DL on raw image data or on human interpretable reconstructed images. These methods (data augmentation using GANs/style transfer) have emerged to provide efficient solutions to translate images across various domains to harmonize images rather than the radiomic features. It would be worthwhile to harmonize raw image data with underlying hidden information from the scanners rather than using human interpretable reconstructed images. Studies have been conducted to show that performance of models on raw image data is at par with that of reconstructed images [56]. Furthermore, GANs have shown promising results to overcome the multi-centric variation. However, GANs are arduous to train due to vanishing gradient challenges that can completely stop the learning process. They are data hungry and suffer from mode collapse causing them to generate similar looking images. On the other extreme, they can also add unrealistic artefacts in the images. Moving forward, advances in the field of style transfer may prove useful to harmonize images without the need of scanner-specific radiomic models. Neural style transfer and its derivatives could extract texture information [101] which could be very useful to obtain reproducible radiomic features in multi-centric trials. Although these techniques have not been specifically analyzed to improve radiomic feature reproducibility, it can be worthwhile to extend their potential to radiomic features.

In the feature domain, various methods have been implemented directly on radiomic features to evaluate its reproducibility and its generalizability across scanner protocol settings. The most convenient and comparatively easy way is to identify reproducible features and focus explicitly on them to evaluate the model’s performance. The selection of reproducible features helps build robust models, yet one drawback is that several informative and useful features might be excluded for analyses while extracting ‘reproducible features’. Furthermore, there is no generalized threshold for all features above which the latter can be labelled as ‘reproducible enough’, the condition to be met is that the signal is stronger than the noise. These studies report that variation in scanner acquisition and reconstruction parameters have an impact on the radiomic features and their reproducibility hence highlighting the importance of utilizing harmonization methods for stabilizing radiomic features under analysis. Normalization techniques such as min-max normalization, z-score normalization, histogram matching for intensities, and ComBat harmonization have been explored for radiomic studies. Basic statistical approaches (rescaling/standardization) might be too simplistic to apply considering the fact that some image modalities are complex and non-linear (MRI). Histogram matching or equalization is an efficient method to normalize the intensity scales of images, but it is often used as a pre-processing step to ‘clean’ the data before feeding it to the radiomics models. On the other hand, ComBat tries to get rid of the ‘batch effects’ (or scanner/protocol variability) by shifting data distributions while also preserving the biological variation in the data under analysis. However, ComBat relies heavily on labelled data to perform efficient batch correction and estimation [40]. Another disadvantage is that if new data is to be harmonized then it must be added in the existing pool of data for ComBat to perform correctly. Alternatively, normalizing ‘deep’ features [21] can also be an efficient way to improve the reproducibility of features since DL has a wide scope with various architectures and techniques. Domain adaptation techniques using DL and GANs have the ability to translate images from one domain to another and can thus increase the overlap of feature distribution between two unharmonized images. Data augmentation, adversarial training, and normalization techniques in combination with neural networks could complement the benefits of neural network training.

Furthermore, to assess the effects that image acquisition parameters have on radiomic features studies have been conducted on phantom images or on images acquired from several different patients to reduce the dosage exposure given to individual patients. One issue with images acquired from different patients is that it introduces high variability due to differences in patient positioning and anatomy [146,188]. On the other hand, objects used for phantom studies are easy to scan for multiple test-retest studies and can be conveniently transported between various imaging sites. Additionally, instructions/guidelines could be set for standardizing the image acquisition parameters to control its variability, tailored to fit the clinical practice. Pre-processing raw sensor-level data is an interesting approach to harmonize images if one wants to make use of the latent information within these raw images. Since a lot of research has already been done using ComBat methods, it would be worthwhile to apply deep learning solutions such as GANs, style transfer, or even normalization using deep learning techniques. These deep learning solutions need further research to show their true potential by applying them to more real medical datasets.

Radiographic phantoms are not the true representatives for realistic patient tissues and this is proved by Mackin et al. [27] who conducted a study showing that the radiomic features extracted from NSCLC (non-small cell lung cancer) and the same features extracted from a phantom (made up of 10 different materials) did not yield the same values for any of the features [27]. Besides, acquisition and reconstruction parameters have also proved to have effects on the radiomic features [20,127,135,189]. Different vendors may have different reconstruction methods and reconstruction parameters that are tailored accordingly at each site/institution.

## 5. Conclusions

Radiomics is an emerging field and standardization of radiomic features and/or images is crucial for its survival and impact in this domain when it comes to multicentric studies. Various harmonization methods have been investigated to assess the reproducibility and validation of radiomics across different scanners and protocol settings. This review has covered various topics ranging from methods in the image domain (GANs, style transfer, and regulations guidelines) to methods in the feature domain (statistical normalization, identification of reproducible features, ‘deep’ feature normalization). The use of harmonization methods has the potential to be beneficial in multi-center studies and the reproducible radiomic features can be practically useful in the decision- making process. Style transfer techniques, with style/content loss or cycle-consistency loss (e.g., cycleGAN) or in combination, have the potential to harmonize data in the image domain, despite the limitations of GANs. Style transfer needs just two images to work without any prior details about scanners/protocols and hence could be applied on old images in retrospective studies and on unpaired images. However, in context of harmonizing images, a limited number of experiments have been conducted and even less for radiomic studies. For harmonization of radiomic features, ComBat methods seem to be extensively used, although normalizing features using deep learning techniques (e.g., domain adaptation methods) can be the way to go ahead too. [21] Showed that normalization using DL can be extended to images coming from unknown scanners and it would be worthwhile to apply this method in combination with GANs in future directions. More work is still needed on identifying limits of features extracted and normalization methods based on just how different the produced images are. Differences linked to scanner model, slice thickness, or reconstruction kernel will likely be in clusters where close clusters can be more easily compared than clusters that are far away from each other. Large datasets of phantom and test-retest data need to be collected for this purpose.

## Figures and Tables

**Table 1:** Summary table of standardization guidelines/regulations set for image acquisition and reconstruction parameters across various centers.

Standardization of Image Acquisition and Reconstruction Parameters across Various Centers Related to Clinical Trials			
Reference	Data	Variation Across	Summary
Mottet et al. [42] Cornford et al. [43] (ESTRO)	CT and MRI images (prostate cancer)	NA	Provided guidelines procedures and methods for image-guided radiation therapy (IGRT) in prostate cancer
Boellaard et al. [45] (EARL)	PET imaging	Scan acquisition, image processing, image interpretation	Provides guideline/regulations for oncology
Luh et al. [48] (ASTRO)	NA	NA	Developed a ‘practice parameter’ for IGRT, and provided quality assurance standards, personnel qualifications, indications and guided documentation for imaging
Sachs et al. [51]	CT and MRI images	CT and MRI protocols	Reduced the number of MRI protocols from 168 to 66 and CT protocols from 248 to 97 across scanners and centers by selecting an appropriate clinical-driven protocol and standardization process
Center for Drug Evaluation and Research (FDA) [52]	NA	Image acquisition parameters	Provided guidelines to focus on image acquisition in clinical trials conducted to support authorization of drugs and biological products

**Table 2:** Summary table of post-processing methods of raw sensor-level image data.

Post-Processing of Raw Image Data			
Reference	Data	Variation Across	Summary
Lee et al. [56]	Raw sinogram CT data (head and whole-body)	Acquisition parameters in terms of projections and detector like sinograms	Investigated the performance of a CNN for classifying raw CT data in sinogram-space to identify body region and detect intracranial hemorrhage
Gallardo-Estrella et al. [58]	Reconstructed CT images (emphysema in lungs)	Reconstruction kernels	Proposed a method to reduce variability due to different reconstruction kernels in CT images by decomposing each CT scan into a set of frequency bands and the energy in each frequency band is scaled to a reference value iteratively.

**Table 3:** Summary table of data augmentation methods using GANs.

Data Augmentation Using GANs			
Reference	Data	Variation Across	Summary
Zhong et al. [76]	MRI images (neonatal brains)	Scanners, acquisition protocols	Utilized a dual GAN, with U-Net as the backbone to harmonize the diffusion tensor imaging (DTI) derived metrics on neonatal brains
Modanwal et al. [78]	MRI images (breast)	Scanners	Utilized a cycleGAN to perform intensity harmonization on MRI breast images obtained from two different scanners
Cackowski et al. [79]	MRI images (brain)	Scanners, acquisition protocols	Conducted a comparative study was conducted by Cackowski et al. [79] between ComBat and cycleGAN to harmonize multi-centric MRI images
Guha et al. [63]	CT images (trabecular bone (Tb) microstructures)	Scanners	Conducted a study that transforms low-resolution (LR) CT scans of trabecular (Tb) bone microstructures into high-resolution (HR) CT scans, obtained from two scanners (LR from Siemens FLASH and HR from Siemens FORCE; paired images), using GAN-CIRCLE [80]
Wei et al. [87]	CT images (chest)	Dosage, slices thickness	Utilized a 3D GAN to normalize CT images to classify and detect pulmonary nodules

**Table 4:** Summary table of style transfer methods.

Style Transfer			
**Reference**	**Data**	**Variation Across**	Summary
Gatys et al. [101]	Non-medical images (mostly artistic images)	NA	Utilized a CNN to perform neural style transfer using Gram matrix
Li et al. [104]	Non-medical images (mostly artistic images)	NA	Treated neural style transfer as a domain adaptation problem and proved that matching Gram matrices of filter responses is equivalent to minimizing MMD [105]
Xu et al. [106]	Non-medical images (mostly artistic images)	NA	Combined the concepts of original neural style transfer with the concept of adversarial training for arbitrary style transfer from multi-domain images
Yang et al. [111]	CT images (head, facial bone)	Reconstruction kernels	Investigated the effects of different kernels on CT images and proposed an unsupervised image style transfer method by utilizing a cycleGAN with AdaIN [108] that works on unpaired images
Liu et al. [115]	MRI images (chest)	Multi-center datasets, image acquisition parameters	Harmonized MRI images from multiple arbitrary sites using a style transferable GAN entailing cycle consistency, style and adversarial losses.
Armanious et al. [116]	PET, CT images (brain)	Multi-modal dataset	Developed MedGAN architecture which consists of a cascade of UNet blocks to obtain sharper translated images (CasNet) along with Gatys et al.’s [101] style transfer losses.
Clancy and Milanko [117]	X-rays (chest)	Healthy and unhealthy patients	Utilized the cycleGAN with adversarial losses to perform style transfer.
Fetty et al. [120]	MRI, CT images (pelvic malignancies)	Multi-model dataset	Used StyleGAN with baseline GAN architecture and AdaIN method for transferring style across images.

**Table 5:** Summary table of literature which focused on identification of reproducible features.

Focusing on Reproducible Features (Identification of Reproducible Features)			
Reference	Data	Variation Across	Summary
Shiri et al. [127]	PET/CT phantom	Image reconstruction settings, scanners	Reproducibility and variability of radiomic features were evaluated using the coefficient of variation (COV)
Bailly et al. [129]	PET scans (gastro-entero-pancreatic neuroendocrine tumors)	Multi-centric trials (acquisition and reconstruction parameters)	Analyzed the reproducibility of textural features in PET scans across different acquisition and reconstruction parameters in the context of multi-center trials
Prayer et al. [145]	CT scans (fibrosing interstitial lung disease (fILD))	Scanners, test-retest study	Investigated the inter-and intra-scanner repeatability and reproducibility of computed tomography (CT) radiomic features (radiomic feature) of fILD
Lee et al. [149]	MRI scans (phantom, brain lesions)	Scanners, scanning protocol	Investigated the reproducibility of MRI radiomic features across different MRI scanners and scanning protocol parameters
Peerlings et al. [150]	MRI scans (ovarian cancer, colorectal liver metastasis)	Vendors, field strengths	Extracted stable parametric MRI radiomic features with a minimum concordance correlation coefficient of 0.85 between data derived from 61 patients’ test and retest apparent diffusion coefficient maps

**Table 6:** Summary table of basic statistical approaches.

Statistical Normalization			
Reference	Data	Variation Across	Summary
Chatterjee et al. [160]	MRI images (endometrial cancer)	Multi-center datasets	Investigated the effect of applying rescaling and standardization as normalization transformations in MRI images obtained from two different institutes. These transformations were applied separately on balanced training and testing sets rather than applying normalization for the entire dataset
Haga et al. [161]	CT images (non-small cell lung cancer (NSCLC))	Multi-centric datasets	Used z-score normalization to standardize the radiomic features extracted from CT images of NSCLC patients from The University of Tokyo Hospital and TCIA (the Cancer Imaging Archive)

**Table 7:** Summary table of intensity harmonization methods.

Intensity Harmonization Techniques			
Reference	Data	Variation Across	Summary
Crombé et al. [162]	MRI images (sarcoma)	Multi-centric datasets	Performed IHT (standard normalization, z-score normalization, standardization per signal intensities of healthy tissue, histogram matching and ComBat harmonization) as a post-processing method on T2-weighted MRI images of sarcoma patients to enhance the MFS (metastatic-relapse-free survival) predictive models
Masson et al. [166]	Contrast enhanced CT images	Multicenteric dataset	Applied histogram equalization (enhancing the contrast by flattening the histogram) on images to normalize intensity scales to pre-process images prior to applying ComBat harmonization method on top of it.

**Table 8:** Summary table of ComBat methods and its derivatives.

ComBat Method and Its Derivatives			
Reference	Data	Variation Across	Summary
Fortin et al. [169]	DTI data	Acquisition and reconstruction parameters	Used ComBat to harmonize cortical thickness measurements calculated on DTI data to remove variations in feature values attributed to differences in acquisition and reconstruction parameters.
Orlhac et al. [170]	CT scans(phantom [180], lung cancer)	Multi-centric dataset	Investigated the potential of ComBat to correct for the variations of CT radiomic features extracted from scans collected from different centers.
Orlhac et al. [171]	PET scans	Acquisition parameters	Investigated the potential of ComBat to harmonize radiomic features extracted from PET scans acquired differently
Ibrahim et al. [174]	Phantom CT [176]	Acquisition and reconstruction parameters	Proposed a framework that guides the use of ComBat in radiomics analyses to assess the performance of ComBat
Ibrahim et al. [177]	Phantom CT [176]	Imaging protocols, vendors	Investigated the reproducibility of radiomic features in a pairwise manner and performed ComBat harmonization on it.
Ibrahim et al. [178,179]	Phantom CT	In-plane resolution	Performed pairwise comparisons between the scans and reported that radiomic features are affected differently by the degree of variation within a single reconstruction parameter (in-plane resolution).

**Table 9:** Summary table of normalization methods using deep learning techniques.

Normalization Using Deep Learning			
Reference	Data	Variation Across	Summary
Andrearczyk et al. [21]	Phantom CT [27]	Acquisition and reconstruction parameters	Proposed a DL-based technique trained on phantom data to normalize various types of features including hand-crafted and deep features using a simple neural network to learn a non-linear normalization transformation
Rozantsev et al. [181]	Non-medical images	Synthetic, real image domains	Adapted divergence-based approaches for domain adaptation by using a two-stream CNN architecture (one in the source domain with synthetic images and the other in the target domain with real images) with unshared weights
Sun and Saenko [182]	Non-medical images	Different image domain in Office dataset [184]	Adapted divergence-based approaches for domain adaptation by using DeepCORAL [183] architecture
Dinsdale et al. [187]	MRI images (neuro)	Multi-centric dataset	Adapted the framework as in [186] and utilized an iterative update approach that aimed to generate scanner-invariant (i.e., harmonized features) representations of MRI neuroimages while evaluating the main task (segmentation).

## Data Availability

No new data were created or analyzed in this study. Data sharing is not applicable to this article.
